# Modulation of the lens water content changes the stiffness of the *ex-vivo* non-decapsulated bovine lenses

**DOI:** 10.3389/fopht.2025.1676751

**Published:** 2025-10-10

**Authors:** Chen Qiu, Dingchang Shi, Xingzheng Pan, Yadi Chen, Paul J. Donaldson

**Affiliations:** Department of Physiology, School of Medical Sciences, Aotearoa New Zealand National Eye Center, Faculty of Medical and Health Sciences, University of Auckland, Auckland, New Zealand

**Keywords:** lens, MRI imaging, water content, spin test, shear modulus, presbyopia

## Abstract

**Purpose:**

To determine whether modulation of lens water content can alter the stiffness of the *ex vivo* bovine lens which have a similar stiffness profile to the presbyopic human lens.

**Methods:**

Bovine lenses cultured in isotonic artificial aqueous humor (AAH) were initially subjected to either MRI imaging using a clinical 3T scanner or a spin test to obtain baseline measurements of water content and shear modulus, respectively. Lenses were then exposed to either hypotonic or hypertonic stress to swell or shrink lenses, respectively, or isotonic AAH + ouabain or high extracellular potassium (AAH-High-K^+^) to inhibit lens water transport, for up to 4 hours before repeating the MRI scans and spin test.

**Results:**

In isotonic AAH both free and total water was higher in the outer cortex of the lens relative the central lens nuclear region, but the shear modulus profile had the opposite profile being highest in the lens nucleus. Exposure to hypertonic AAH that shrinks the lens caused a loss of lens water and an increase in the shear modulus in the lens nucleus that served to steepen the shear modulus profile. In contrast, exposure to hypotonic-AAH to sweel the lens increased both free and total water content through all regions of the lens and caused a reversal of the shear modulus so that the nucleus of the lens became less stiff than the outer cortex. These effects of osmotic stress on the shear modulus profile were partially reversed upon the return of lenses to isotonic AAH. Inhibiting lens water transport under isotonic conditions caused more subtle increases in lens water content than seen with hypotonic challenge but still cause a similar softening of the nucleus but had no major effect on the shear modulus in the outer cortex of the bovine lens.

**Conclusions:**

Our results demonstrate a link between lens water content and the stiffness of the nucleus of the bovine lens. This suggests that the modulation of lens water transport represents a novel strategy for the development of pharmacological interventions designed to restore accommodation in presbyopes by softening of the nucleus of the human lens.

## Introduction

Our sense of sight is critically dependent on the ability of the lens to focus light onto the retina. The optical properties of the lens are in turn the product of its transparency and refractive properties, which are both determined by lens tissue architecture and cellular function (1). Although not the major contributor to optical power (2), the ability of the lens to dynamically alter its shape enables the eye to change its point of focus, or accommodate (3). Presbyopia is the loss of near visual function that results from the gradual loss of accommodative amplitude with age (4). Symptoms of presbyopia commence in human emmetropes with normal visual acuity at around 40–50 years of age. This age-related loss of visual performance requires both distant and near vision correction, and globally it is estimated that in 2020, some 2.1 billion people were affected by presbyopia (5, 6). The most widely accepted theory for the onset of presbyopia is an age-related increase in the stiffness of the central nucleus of the lens relative to the more peripheral outer cortex of the lens (7). However, the nature and causes of this increase in nuclear stiffness remain elusive, although most current theories have attributed this age-dependent regional increase in lens stiffening to changes in the mechanical properties associated with increased sclerosis of the fibre cells that make up the bulk of the lens (4, 7, 8).

In this study we proposed to test a new theory - that the increase in lens stiffness is driven by an age-dependent decline in the ability of the lens to control the water content (9, 10). It has been known for many years that the free water content of the human lens increases with age (10, 11), however, the relevance of lens water content to the onset of presbyopia has only recently become apparent (9). Using MRI to image water content in animal lenses we have recently shown that the water is actively removed from the lens by a microcirculation system (1, 12), which others have shown generates a significant intracellular hydrostatic pressure gradient in the lens nucleus (13, 14). Applying these MRI-based approaches to human subjects we have shown that an increase in the free water content in the anterior region of the human lens contributes to the change in power observed in young subjects undergoing accommodation, while in older presbyopic participants the application of the same accommodative effort produced a decrease in free water content (9, 15). In addition, to these dynamic changes in free water content associated with the loss of accommodative capacity, we also observed an increase in free water content but not total water in the nucleus of human lenses with age (10). Since water exists in biological tissues as either free water or water bound to proteins (16, 17), changes in free water that occur in the absence of changes in total water content must reflect changes in the amount of water bound to lens proteins in the lens nucleus. These observations have led us to hypothesize that changes to both the dynamic and steady state regulation of lens water content maybe an underlying cause of an increase in nuclear stiffness observed in the older presbyopic lens.

To test this theory, parallel measurements of lens water content and stiffness performed on intact lenses before and after exposure to protocols designed to alter lens water transport are required. To achieve this, we have developed an experimental workflow that utilises MRI protocols optimised to measure water content in multiple bovine lenses (12, 18–20), and have introduced a spin test (21–24) to measure how the stiffness profiles of bovine lenses are affected by modulation of lens water transport. Using this dual approach we have performed proof of principle experiments that show altering the water content of the lens via either osmotic challenge or inhibiting lens water transport can alter lens stiffness in different regions of the bovine lens.

## Materials and methods

### Lens culture conditions

Fresh bovine eyes were obtained from a local abattoir (Auckland Meat Processors, Auckland, New Zealand) and immediately transferred to the laboratory. Lenses were extracted from the eyes by cutting the zonules and the removal of the adherent vitreous humour, before being transferred using a sterile plastic spoon to individual chambers of a 12-well tissue culture plate that contained isotonic artificial aqueous humor (AAH) pre-warmed to 37°C (**Table 1**). Lenses were placed in an incubator for up to 1 hour to assess for any change in transparency, and any lens whose transparency appear compromised was excluded from further analysis. Transparent lenses were then allocated for analysis by MRI or the spin test system to collect baseline measurements of lens water and stiffness, respectively. Lenses were then placed in solutions designed to change the water content of the lens by either altering the osmolarity or inhibiting the lens microcirculation system (**Table 1**) and returned to the incubator for up to 4 hours before repeating the MRI and spin test measurements.

**Table 1:** Composition of different AAH solutions.

Component	Isotonic AAH	Hypotonic AAH	Hypertonic AAH	High K+ AAH	Ouabain-AAH
NaCl (mM)	125	76.8	173.2	25	125
KCl (mM)	4.5	4.5	4.5	100	4.5
MgCl_2_ (mM)	0.5	0.5	0.5	0.5	0.5
CaCl_2_ (mM)	2	2	2	2	2
NaHCO_3_ (mM)	10	10	10	10	10
Glucose (mM)	5	5	5	5	5
Sucrose (mM)	20	20	20	20	20
HEPES (mM)	10	10	10	10	10
Osmolarity (osmol/L)	300	220	410	300	300
pH	7.1	7.1	7.1	7.1	7.1
Ouabain (mM)					1

### Measurements of lens geometry and volume changes

Bovine lenses before and after treatment were transferred and positioned on a customized lens holder, machined from a thin stainless-steel sheet with the anterior surface facing upwards. Lenses were back lit using a diffuse light source (Dell model 1908FP LCD screen) to enhance the visualization of the lens edges and sagittal images captured using a digital SLR camera (Canon EOS 1100D, Tokyo, Japan) fitted with a Canon 50 mm f1.8 lens. Lens equatorial diameter (ED) and lens thickness (LT) were extracted for each lens volume was estimated by computing the solid revolution of the 2D image along the optical axis using the following discrete integration formula (25):

(1) $$V=\pi \int_{-T_{P}}^{T_{A}}{\left[h(x)\right]}^{2}dx$$

where *T_A_* is the anterior lens thickness, *T_P_* is the posterior lens thickness, *h(x)* is a higher-order polynomial describing the lens surface at a given location *x*.

### MRI measurement of lens water content

#### Experimental protocols

Up to 12 transparent bovine lenses maintained in AAH were placed into individual wells of a custom-designed sample holder that contained a 5mm deep peripheral water channel that surrounded the inner region of the chamber. This chamber was designed to minimise susceptibility mismatch artefacts caused by the interface between the holder and the surrounding air. The sample holder containing the organ cultured lenses was then placed in a 16-channel hand/wrist coil (Siemens, Germany), which was in turn positioned in a 3T clinical MRI scanner (MAGNETOM SKYRA, Siemens Healthcare, Erlangen, Germany) located in the Centre for Advanced MRI (CAMRI) at the University of Auckland. Lenses incubated in AAH were then scanned using an established clinical T1 mapping protocol that utilized volumetric interpolated breath hold examination (VIBE) sequences with dual flip angles. The flip angels (*a*) chosen were 4° and 23 ° (9, 10), and the imaging resolution was 0.2 × 0.2mm in-plane with a slice thickness of 2mm. Other imaging parameters include time of echo (TE): 2.49ms; time of repetition (TR): 15ms; parallel imaging with acceleration factor of 2. T1 and PD values are calculated using our customised working flow (10). After an initial baseline scan in AAH the bathing media was changed to one of the test solutions (**Table 1**) and returned to an incubator for up to 4 hours before rescanning the lenses.

#### Data analysis

The low-resolution B1 map was resliced and co-registered with one of the volumetric sets ( a = 23°). The pixel-wise correction was then performed to correct the signal biased by field inhomogeneity (26) using Equation 2:

(2) $$\;\;\begin{array}{c} \frac{S}{sin(\alpha b_{1})}=\frac{S}{cos(\alpha b_{1})}e^{-\frac{T_{R}}{T_{1}}}+PD(1-e^{-\frac{T_{R}}{T_{1}}}) \end{array}$$

Where S is the signal intensity with respective flip angle a, $b_{1}$ is a multiplier that denotes the ratio of actual α (biased by the field inhomogeneity) and ideal α, which is calculated from the acquired B1 map (27). PD denotes the proton density before excitation. In this case, it denotes the total water protons that generate the MRI signal in the lens. After the signal correction, T1 and PD maps were obtained from linear fitting. PD of the water, $PD_{water}$ was calculated from the pure water, which served as a reference to calculate the tissue water content. The overall water content of the tissue, ${\text{ρ}}_{\text{tissue}}\;$ is determined by Equation 3:

(3) $${\text{ρ}}_{\text{lens}}=\;\frac{PD_{lens}}{PD_{water}}\;$$

Data fitting was performed using custom-written routines in MATLAB (MathWorks, Natick, MA, USA). A one- dimensional trend analysis was performed for the resultant T1 & PD maps of each bovine lens, consistent with our previous lens MRI studies. For this, T1 and ${\text{ρ}}_{\text{lens}}$ values over a 5-pixel-wide band along the lens equatorial axis were extracted and averaged, and plotted against the normalised distance, *r/a* along the axis (10, 20).

### Spin test measurement of lens stiffness

To measure lens stiffness, we have adopted the approach first introduced by Fisher et al., that spins the lens to estimate relative changes in the entire internal stiffness profile (shear modulus) of decapsulated lenses (11, 22). To achieve this we have implemented an improved iteration of a spin test system developed by Burd et al., (21) and have used it to investigate how modulation of lens water content affects the shear modulus in different regions of lenses spun with their capsule attached.

#### Experimental protocols

Transparent lenses, free from adherent tissue from the ciliary body that could influence the geometry of the spun lens, were removed from the incubator and placed in a holder that supports the lens during the spinning protocol. The correct vertical and horizontal alignment of lenses in the holder was first checked by videoing each lens as it is slowly rotated (~5 RPM) to visually assess for tilt, and then manually altering the lens position to remove/minimise any observed lens tilt. Once aligned lenses were cycled through an acceleration and deceleration to remove any extra fluid from the surface of the lens, since the presence of excess fluid obscured the true outline of the lens. A pair of images, before (stationary) and during spinning (deformed), were then collected for each lens incubated in AAH. The time taken to align a lens in the holder and subsequently collect a pair of images was normally between 5 to 10 minutes, which minimised the time each lens was exposed to air and minimised any potential changes to lens stiffness due to variations in lens hydration (24). At the end of a spin test lenses were immediately returned to the incubator and then placed in either AAH or a test solution (**Table 1**) for up to 4 hours before repeating the spin test. Thus, for each test solution two sets of data were obtained, with each set consisting of one stationary image and one ‘forced’ deformed image, that can then be independently analysed for changes in stiffness.

#### Spin test system

Individual lenses are placed on a holder, manufactured to an accuracy ±0.1 mm using a 3D resin printer. The holder was in turn attached to the shaft of a rotary motor (Model BLDC, Maxon Pacific, NSW, Australia), by a rigid coupler to eliminate aby misalignment artefacts. The precession error was approximately 0.03mm which is in the same order of magnitude as the radial play of the rotary motor of 0.012mm. The operation of the rotary motor and its controller plus three light-emitting diodes (LED’s), and a camera (Blackfly^®^ S BFS-U3-50S5M, Voltrium Systems PTE LTD, Singapore) fitted with a lens were coordinated by a microcontroller (Uno WiFi REV2, Arduino, Italy). The rotational speed, acceleration and deceleration of the motor plus the motor position (4096 encoder counts per full 360° rotation) were fed into the microcontroller which in turn controlled the LED illumination and camera activation. Camera exposure time was balanced through trial-and-error with the amount of light available, which is determined by the number of LED flashes per lens rotation, to produce correctly exposed images. Images of the stationary lens were collected with the LED’s constantly on and optimised camera parameters (gain = 2.0 dB, gamma = 0.3, exposure time = 498 μs, black level = -2%). For the spun lens, image acquisition parameters of the camera were: gain = 2.0 dB, gamma = 0.3, black level = -2% and exposure time = 4 s, and the LED’s were programmed to flash every 0.5236 radians (30 encoder counts/360° * 2) so that 12 different positions of the lens are captured and averaged during the long exposure time in the acquisition of the spun lens geometry.

#### Extraction of lens geometry

**Figure 1:**
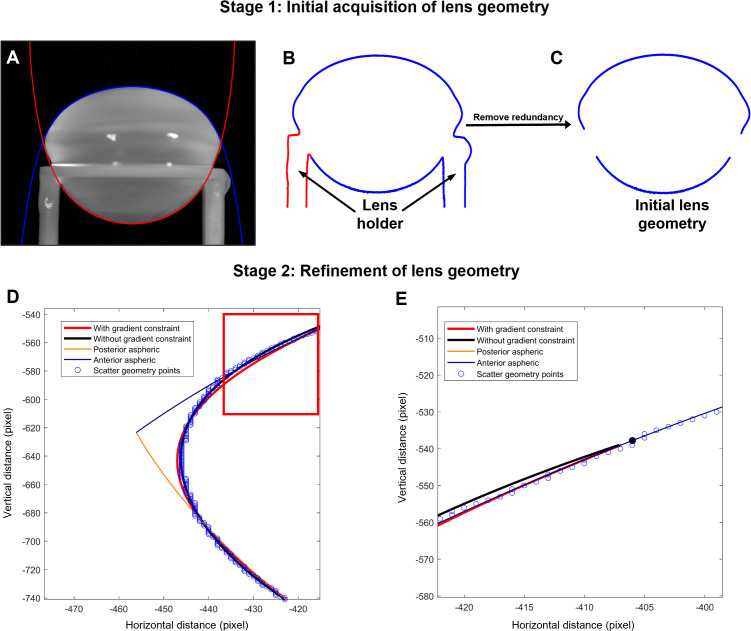
Extraction of lens geometry. **(A)** Image of a bovine lens sitting on the stage that holds the lens in place during the spinning protocol. The anterior (blue) and posterior (red) surfaces of the lens are fit with the aspheric lens equations. **(B, C)** Image segmentation was initially applied to capture the outline for the lens in the holder **(B)**, before manually removing the outline of the holder **(C)**. **(D)** Zoomed in area of the lens geometry (blue points) near the equator and vertex showing the initial anterior (blue line) and posterior (orange line) aspheric fits plotted against horizonal and vertical distance in pixels from the optical centre of the lens. Modification of initial fit of the segmented image with the aspheric lens equations is performed without (black line) and with (red line) gradient restrains to accommodate for the abrupt change in geometry of the lens at the equator that is not accurately captured by the connecting vertices of the anterior and posterior aspheric surfaces. **(E)** Image of the fit from the areas shown in the box in panel D, showing the continuous gradient imposed (red line) to optimise the fit and avoid abrupt changes in the connecting vertices of the aspheric surfaces and the smoothed equatorial geometry estimation.

Image segmentation of images of both the stationary and spun lenses was performed using MATLAB’s image segmenter toolbox (**Figure 1**). GraphCut segmentation with user defined foreground and background seeds were used, and if required, refined using active contours. During post-segmentation of the lens shape the lens regions near the support rings were manually brushed (**Figure 1B**) and removed (**Figure 1C**) to reduce the complexity of modelling the local deformations induced by the support ring (21). Segmented geometry points were then divided into anterior (**Figure 1A**, blue) and posterior groups (**Figure 1A**, red), split at the equator, and then independently fitted to the aspheric lens Equation 4 with three aspheric terms using the curve fitting toolbox [EzyFit 2.44 by Frederic Moisy (28)].

(4) $$Z_{r}=\frac{r_{2}}{R(1+\sqrt{1-\frac{(1+\kappa )r^{2}}{R^{2}}})}+\alpha_{4}r^{4}+\alpha_{6}r^{6}+\ldots$$

Where Z(r) is the magnitude in the z direction, r is the distance from the axis, R is the radius of curvature, κ is the conic constant and $\alpha_{i}$ are coefficients describing the deviations of the surface from axially symmetric quadric surfaces specified by κ and R.

A small rotation is automatically applied to the raw data points to ensure optimal fitting and symmetry due to the symmetric definition of the aspheric lens equation. To accommodate the abrupt change in geometry at the equator, where the connecting vertices of the anterior and posterior aspheric surfaces meet, a smoothed equatorial geometry was estimated (**Figures 1D, E**). This requires the connecting vertices to have the same gradients and hence a continuous gradient must be imposed to avoid abrupt changes in the connecting vertices of the aspheric surfaces and the smoothed equatorial geometry estimation. Such constraint on the fitting was achieved by first computing the numerical gradient at the vertex points using Equation 5:

(5) $$\frac{dz_{v}}{dr}=\frac{z(r_{v})-z(r_{v-1})}{dr}$$

Then by constructing a constraint matrix, using a third order polynomial (Equation 6) with it's first order derivative (Equation 7):

(6) $$z=ar^{3}+br^{2}+cr+d$$

(7) $$\frac{dz}{dr}=3ar^{2}+2br+c$$

(8) $$A_{eq}=[\begin{array}{cccc} 3r_{v1}^{2} & 2r_{v1} & 1 & 0\\ 3r_{v2}^{2} & 2r_{v2} & 1 & 0\\ r_{v1}^{3} & r_{v1}^{2} & r_{v1} & 1\\ r_{v2}^{3} & r_{v2}^{2} & r_{v2} & 1 \end{array}][\begin{array}{c} a\\ b\\ c\\ d \end{array}]$$

(9) $$b_{eq}=[\begin{array}{c} {\frac{dz}{dr}}_{v1}\\ {\frac{dz}{dr}}_{v2}\\ z_{v1}\\ z_{v2} \end{array}]$$

Now computing the objective which is straightforward for all the scattered points:

(10) $$C=[\begin{array}{cccc} r_{p1}^{3} & r_{p1}^{2} & r_{p1} & 1\\ r_{p2}^{3} & r_{p2}^{2} & r_{p2} & 1\\ \vdots & \vdots & \vdots & \vdots \end{array}][\begin{array}{c} a\\ b\\ c\\ d \end{array}]$$

(11) $$d=[\begin{array}{c} z_{p1}\\ z_{p2}\\ \vdots \end{array}]$$

The minimization problem (Equations 10, 11) was solved using least squares constrained by linear equality constraints (Equations 8, 9) to yield a continuous and smooth equatorial geometry (**Figures 1D, E**).

#### Finite element modelling to extract the shear modulus

**Figure 2:**
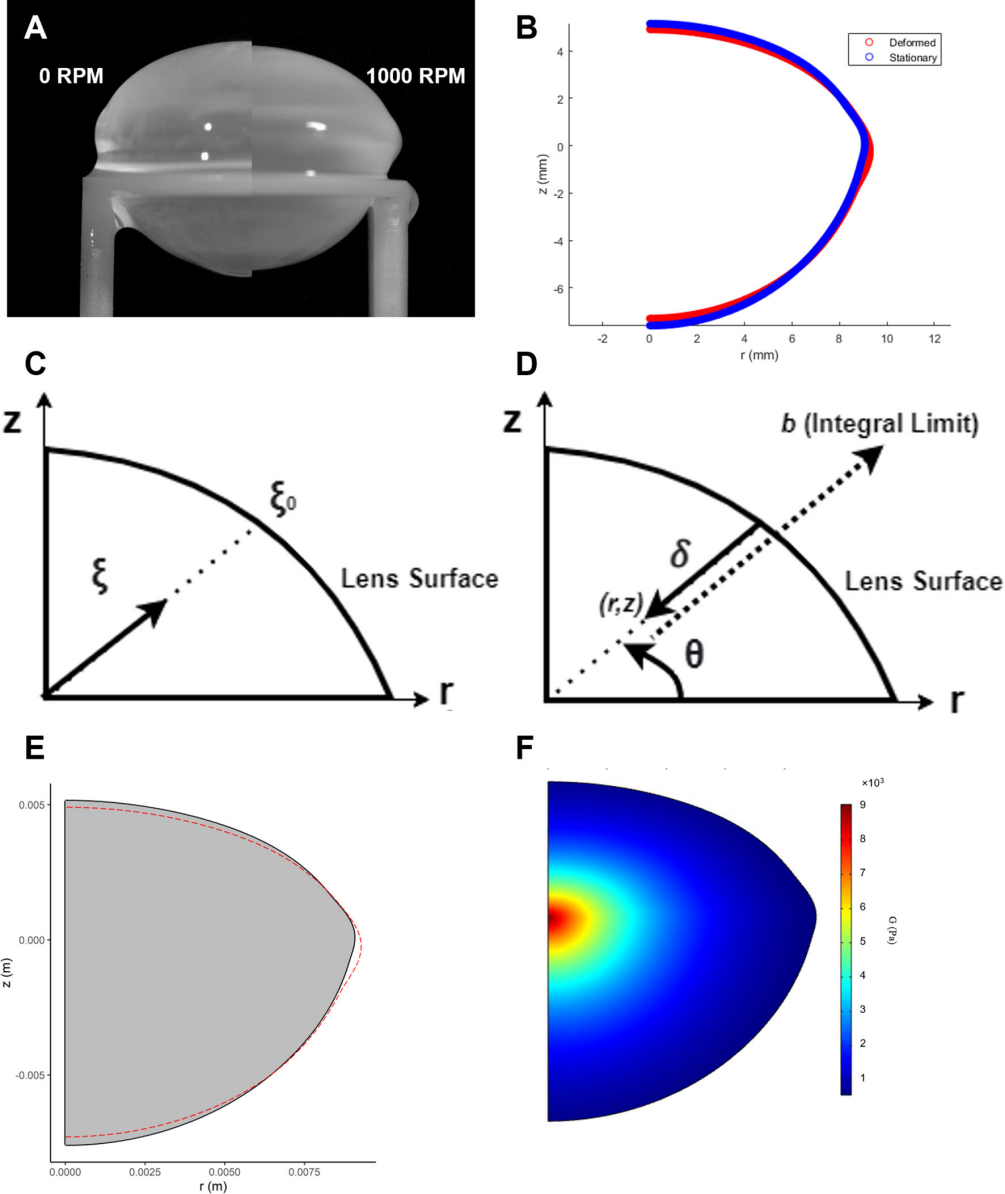
Estimation of the shear modulus in spun bovine lenses using finite element modelling. **(A)** Images of a bovine lens before (0 RPM) and after spinning at high angular velocity (1000 RPM) to induce a deformation. **(B)** Comparison of the extracted geometries from a stationary (blue) and spun (red) representative bovine lens that has been cropped through the optical axis. **(C)** Dimensionless radius expressed in terms of the parameter ξ that complements the spatial variation function of the shear modulus in Equation 12. **(D)** Analogous definition of dimensionless radius in COMSOL. Expression for distance from point (r, z) to the surface, δ. **(E)** A solid mechanics model is used to back calculate the shear modulus of the lens in COMSOL. In this model, COMSOL internally optimizes mechanical parameters (α and β, from Equation 12) to fit the measured lens geometry. The lens is virtually “spun” around its optical axis, and the simulation checks iteratively whether α and β can reproduce the observed change in the initial lens geometry (grey solid) to its deformed (red line) geometry under the applied experimental rotational forces. **(F)** The resultant estimated shear modulus profile generated by finite element modelling of the deformation induced by spinning a bovine lens visualised as a 2D colour map.

To estimate the shear modulus distribution within the crystalline lens a finite element model was constructed using COMSOL Multiphysics (Technic Pty Ltd, TAS, Australia). The model takes as input both the stationary and spun geometries of the lens (**Figures 2A, B**), along with literature-based initial estimates of key material properties such as Poisson’s ratio (ν) and density (ρ) of the lens fiber cells (**Table 2**). We assumed a linear-elastic, isotropic material model and introduced a spatial variation function (SVF) to describe how the shear modulus G varies exponentially with distance from the lens center. This function is given by:

**Table 2:** Approximate biomechanical relevant values for the bovine lens.

Parameter	Approximate value	Units
Poisson’s Ratio	0.4^1^	Dimensionless
Angular velocity	104.72 (1000 RPM) ^2^	Radians per second rads
Youngs Modulus (E)	3.70×10^3^ (young bovine) ^3^ 14.00×10^3^ (old bovine) ^3^	Pascals or Nm^2^
Density	1.104×10^3 4^	Kilograms per meter cubed kgm^3^
Average Shear Modulus (G)	1.23×10^3^ (young bovine)* 4.67×10^3^ (old bovine)*	Pascals or Nm^2^
Model Coefficient – 1 G=exp βξ0	17×10^3^ (Young bovine lens) ^+5^ 10.08×10^3^ (Old bovine lens) ^+5^	Pascals or Nm^2^
Model Coefficient – 2 G=exp βξ0	-0.79 (Young bovine lens) ^5+^ -2.02 (Old bovine lens) ^5^	Dimensionless

^1^Values obtained from (29). Most biological tissue is between 0.3-0.5, an average of 0.4 is employed.

^2^Values obtained from (21).

^3^Values obtained from (30).

^4^Values obtained from (31).

^5^Shear modulus are computed from (30) using E = 2G(1+v); Estimate from graph fit.

(12) $$G=\alpha exp\;(\frac{\beta \xi }{\xi_{0}})\;$$

where ξ is the distance from a point within the lens to its midpoint (defined as the intersection of the axial and equatorial axes), and $\xi_{0}$​ is the distance from the midpoint to the surface along the same radial direction (**Figure 2C**). This ratio $\frac{\xi }{\xi_{0}}$​ defines a dimensionless radial coordinate that allows shear modulus to vary smoothly from the lens center to the periphery.

The implementation of this model in COMSOL required a custom construction of the dimensionless radius and the SVF. Due to the layered structure of lens fiber cells, defining the distance to the lens surface ( δ) from any internal point was non-trivial. For each point (*r*, z) in the lens, we extended a line along the radial direction (determined by the angle θ=atan2(z,r) until it intersected the lens boundary (**Figure 2D**). A COMSOL expression operator was employed to evaluate whether a point extended by a given distance b remained within the domain. The integral of these checks over increasing b yielded δ, from which the dimensionless radius could be calculated. With this in place, the spatially varying shear modulus could be fully defined across the lens geometry.

The objective function used for optimization describes the difference between the model-predicted radial displacement and experimental displacement data. Specifically, it minimized the squared difference between the deformed radial positions ( R+u) from experimental and simulated lenses as a function of axial position ( Z+w). This setup allowed for an inverse solution, where experimental deformations were used to infer the best-fit values of the parameters α and β that define the SVF. Optimization was carried out using the SNOPT solver with an optimality tolerance of 1e-3 and a maximum of 1000 iterations, over a constrained parameter space: 1000≤α≤50000 and −3≤β≤3. Importantly, β was allowed to take both positive and negative values to accommodate the possibility of either increasing or decreasing shear modulus with radius, as seen across different ages and species.

Model validation was performed by simulating the forward problem, with known shear modulus distributions being applied to stationary lens geometries, which were then computationally spun using COMSOL’s rotational frame mechanics to generate deformed geometries. These synthetic data were then fed into the inverse problem to recover the original modulus parameters. Accurate recovery of α and β from these simulated cases confirmed the model’s reliability. Additionally, real bovine lenses with physiological or non-physiological modifications were analyzed to extract spatial stiffness distributions. This modelling framework provides a robust, biology-informed approach for estimating the internal shear modulus of the lens, accounting for complex geometries and material variations.

#### Data presentation

To remove inherent variability on the size of bovine lenses used in this study PD, T1 and shear modulus were all plotted against normalised lens distance (*r/a*), where ±1 and 0 represent the outside and centre of the lens, respectively. Similarly, to exclude lens size as a variable in the measurement of shear modulus, measurements were also normalised to the maximum shear modulus observed in the nucleus of AAH treated lenses at the start of each experiment.

## Results

### Measuring lens stiffness in non-decapsulated lenses using the spin test

**Figure 3:**
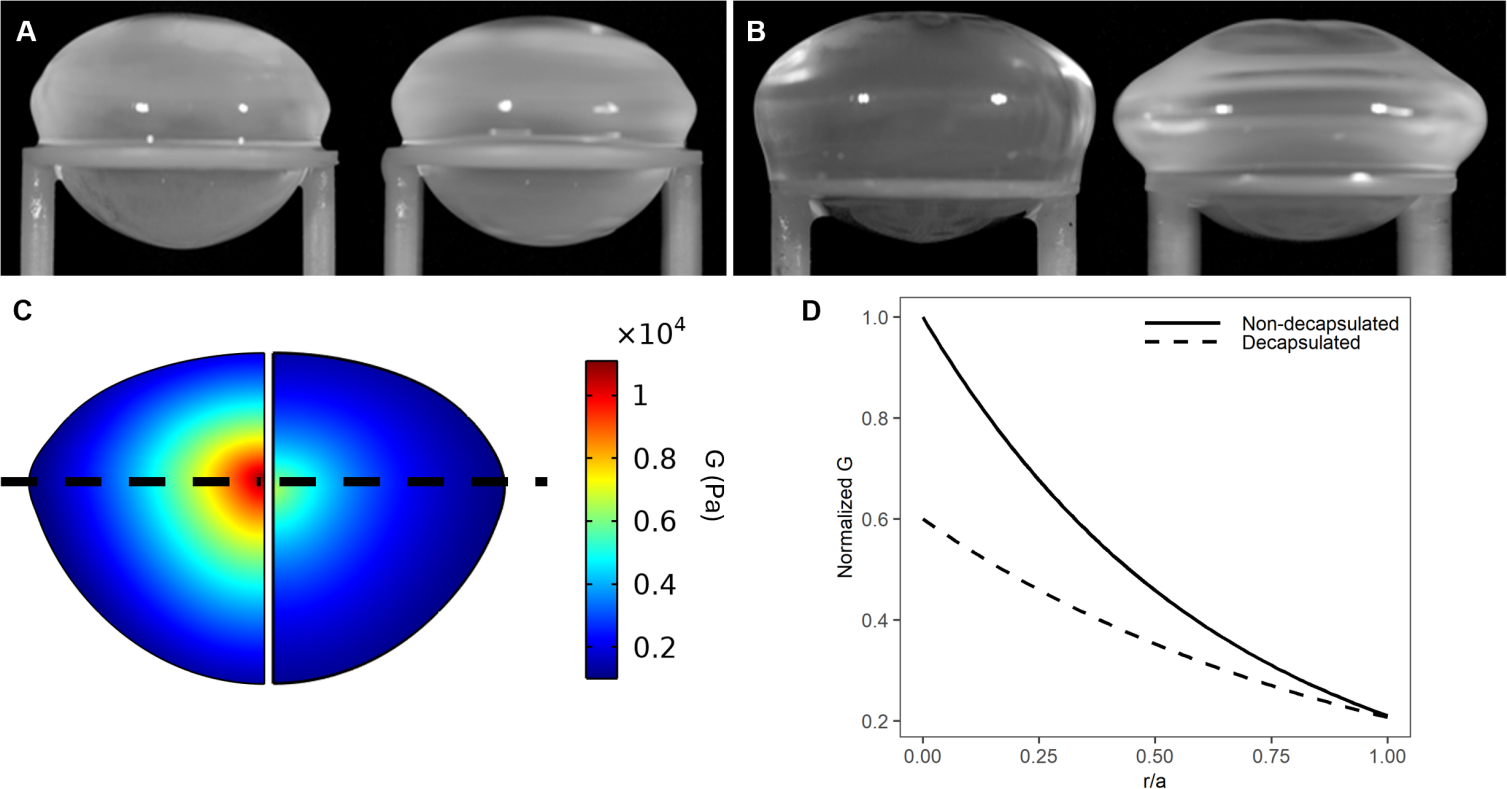
Effect of decapsulation on the shear modulus profile of the bovine lens. **(A, B)** Images of a representative non-decapsulated **(A)** and decapsulated **(B)** bovine lenses before (left) and during (right) spinning at 1000rpm to induce lens deformation. **(C)** Colour maps of estimated shear modulus generated by finite element modelling of the deformation obtained by spinning a bovine lens before (left) and after (right) decapsulation shows that the removal of capsule decreases the estimated shear modulus. **(D)** Representative line profiles of normalised shear modulus plotted against normalised distance into the lens (r/a). Shear modulus has been normalised to the maximum value in the lens nucleus obtained prior to the decapsulating the lens.

Previous studies used a similar spin system to perform shear moduli estimates on the decapsulated porcine lens (21, 24). In these studies, since the primary focus was the measurement of the biomechanical properties of lens fiber cells, the elastic capsule, that normally surrounds and constrains the lens, was removed. Since we wanted to test the effects of experimental perturbations on overall lens stiffness our experiments were performed on lenses that retained their capsule. However, we did some initial experiments to compare the effects of the presence of the capsule on lens deformation and the resultant shear modulus profile (**Figure 3**). Consistent with previous studies it is evident that the geometric deformations are significantly larger in decapsulated lenses (**Figure 3A**) than in lenses spun with their capsule intact (**Figure 3B**). This necessitated the development of robust curve fitting routines (see **Figure 1**) to ensure that the substantially smaller deformations (vertical deformation ~-0.24; radial deformation ~+0.17 mm) could be accurately detected. Consistent with the larger deformation seen in decapsulated lenses, a comparison 2D map of shear modulus showed a steeper shear modulus in the intact lenses relative to the decapsulated lens (**Figure 3C**). Shear modulus line profiles extracted from these maps from multiple lenses were obtained, and the fit parameters α and β obtained for each lens using Equation 12 were averaged and plotted against normalised lens distance (**Figure 3D**). From this analysis it is apparent that the capsule contributes significantly to overall lens stiffness, especially in the lens nucleus. Having shown that we can measure regional differences in shear modulus in intact non-decapsulated lenses, we next wanted to determine whether changing lens water content by exposure to osmotic challenge can alter the shear modulus profile.

### Effects of changing water content on the shear modulus in different lens regions

Since the water content of biological tissues like the lens exists in a dynamical equilibrium between free water and water that is bound to proteins (32), MRI affords the ability to utilise longitudinal relaxation time (T1) values and proton density (PD) measurements to map the distribution of free and total water, respectively, in the different regions of the lens. (12, 20). In the bovine lens both the steady state free and total water contents showed regional differences, with both being lower in the lens core relative to the outer cortex. However, T1 line profiles exhibited a more pronounced parabolic like change than what was observed for total water.

**Figure 4:**
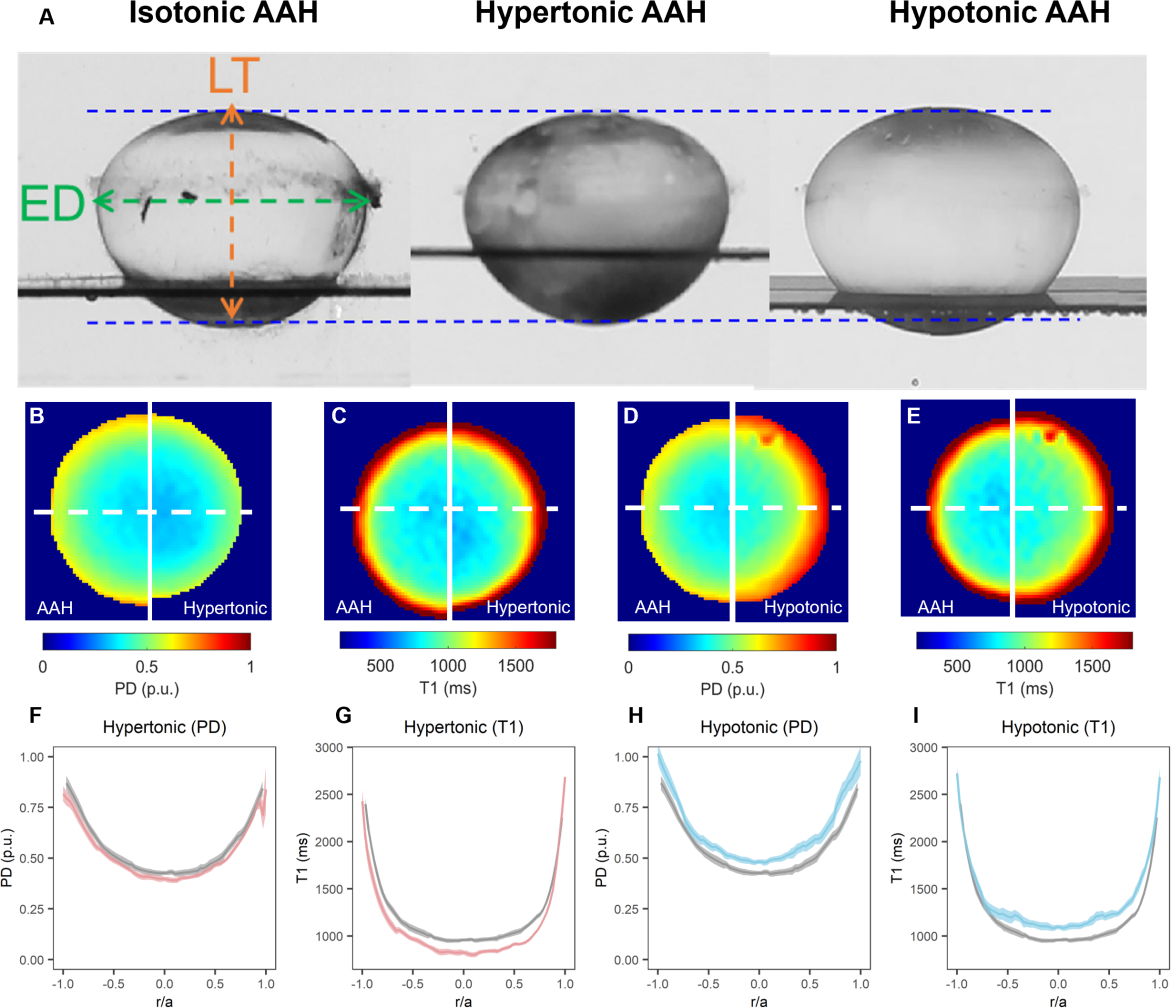
Effect of osmotic challenge on total and free water content in the bovine lens. **(A)** Images showing axial views of bovine lenses incubated in AAH (left), hypertonic (middle) and hypotonic (right) showing the changes in lens thickness (LT) and equatorial diameter (ED) induce by the osmotic challenges. **(B–E)** Colour maps total water (PD, **B, D**) and free water (T1, **C, E**) content from representative lenses before organ cultured in AAH (left panels) and after a 4-hour incubation in either hypertonic (**B, C**, right panels) or hypotonic (**D, E**, right panels) solutions. **(F–I)** Line profiles taken through the optical axis showing the change in total water (PD, **F, H**) and free water (T1, **G, I**) content plotted against normalised lens distance (r/a). Profiles are the average of at least 6 lenses cultured in AAH (grey) followed by a 4-hour incubation in either hypertonic (**F, G**, red) or hypotonic (**H, I**, blue) solutions. Error bars indicating the standard deviation.

Exposure of lenses to hypertonic AAH for 4 hours caused a significant decrease in lens thickness (LT) and equatorial diameter (**Table 3**), which resulted in a 5.85±1.48% decrease in overall lens volume (LV) consistent with a shrinkage of the lens (**Figure 4A**). In contrast, incubation of lenses in hypotonic solution produced a swelling and rounding up of the lens (**Figure 4A**), as seen by a significant increase in LT, and a decrease in ED that manifested as a 3.83±1.19% increase LV (**Table 3**). These physical changes to the geometry caused by osmotic challenge were accompanied by either a decrease (**Figure 4B** & F) or an increase in total water content (**Figures 4D, H**), respectively, across all regions of the lens. In contrast, the change in free water content appear to be more pronounced and localised to the more central regions of lenses exposed to either hypertonic (**Figures 4C, G**) or hypotonic (**Figures 4E, I**) challenge.

**Table 3:** Change in lens geometry and volume caused by osmotic challenge or inhibition of the microcirculation system.

Treatment	Parameter	Before (Mean ± SD)	After (Mean ± SD)	% Change (Mean ± SD)
AAH	LT (mm)	12.489 ± 0.247	12.602 ± 0.397	+0.89 ± 1.19^ns^
	ED (mm)	17.466 ± 0.311	17.368 ± 0.401	–0.56 ± 1.15 ^ns^
	Vol (mm^3^)	1962.36 ± 84.25	1952.81 ± 77.24	–0.47 ± 1.01 ^ns^
Hypertonic	LT (mm)	11.965 ± 0.291	11.743 ± 0.272	–1.86 ± 0.47^**^
	ED (mm)	16.966 ± 0.410	16.455 ± 0.378	–3.00 ± 0.98^**^
	Vol (mm^3^)	1755.31 ± 82.90	1652.65 ± 85.11	–5.85 ± 1.48^***^
Hypotonic	LT (mm)	12.275 ± 0.360	12.881 ± 0.351	+4.95 ± 0.77^***^
	ED (mm)	17.321 ± 0.488	17.054 ± 0.502	–1.54 ± 0.60^**^
	Vol (mm^3^)	1862.72 ± 153.43	1934.97 ± 173.09	+3.83 ± 1.19^***^
High K^+^	LT (mm)	12.404 ± 0.363	12.696 ± 0.259	+2.38 ± 1.07^*^
	ED (mm)	17.118 ± 0.303	17.034 ± 0.284	–0.49 ± 0.18^*^
	Vol (mm^3^)	1842.05 ± 112.14	1888.20 ± 106.50	+2.53 ± 0.51^***^
Ouabain	LT (mm)	12.384 ± 0.289	12.456 ± 0.306	+0.580 ± 0.397^ns^
	ED (mm)	17.126 ± 0.269	16.976 ± 0.237	–0.870 ± 0.292^*^
	Vol (mm^3^)	1831.48 ± 87.06	1807.10 ± 84.14	–1.325 ± 0.910^ns^

Ns, non-significant; *0.01 ≤ p < 0.05; **0.001 ≤ p < 0.01; ***p < 0.001.

**Figure 5:**
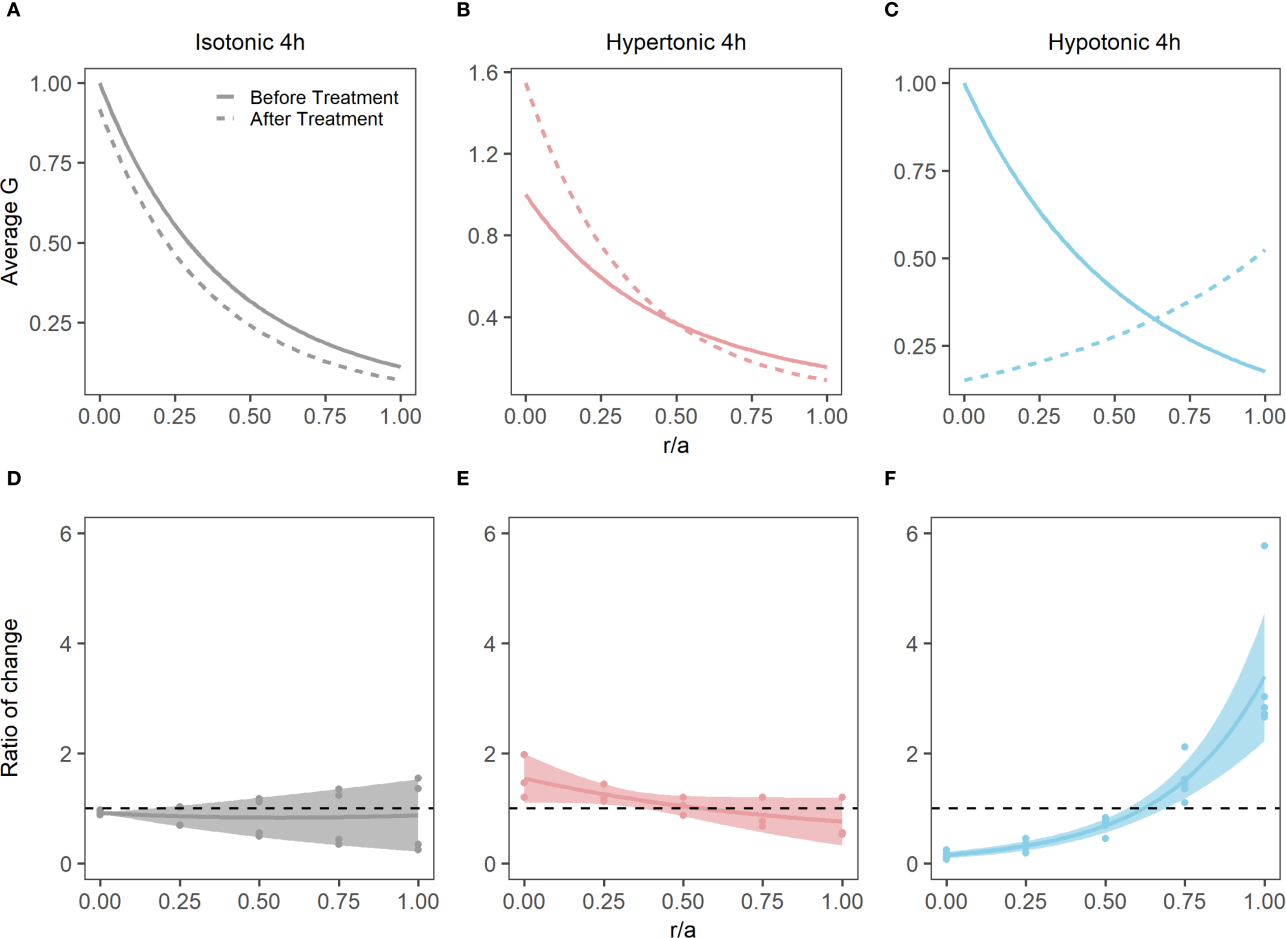
Effect of osmotic challenge on shear modulus profiles in the bovine lens. **(A-C)** Normalised average shear modulus (G) profiles of bovine lenses initially incubated in isotonic AAH (solid lines) and then after (dashed lines) a 4-hour incubation in either isotonic (**A**, n = 4), hypertonic (**B**, n = 3) or hypotonic (**C**, n = 4) AAH. **(D-F)** Spatial comparison of regional lens stiffness (G) across the nucleus-to-periphery axis between baseline and post-incubation conditions for isotonic AAH **(D)**, hypertonic AAH **(E)**, hypotonic AAH **(F)**. The dotted line represents no change between conditions, while the shaded regions represent 95% confidence intervals, and the dots are the biological replicates. In all plots profiles are plotted against normalised lens distance (r/a) where 0 is the centre and 1 is the periphery of the lens.

**Figure 6:**
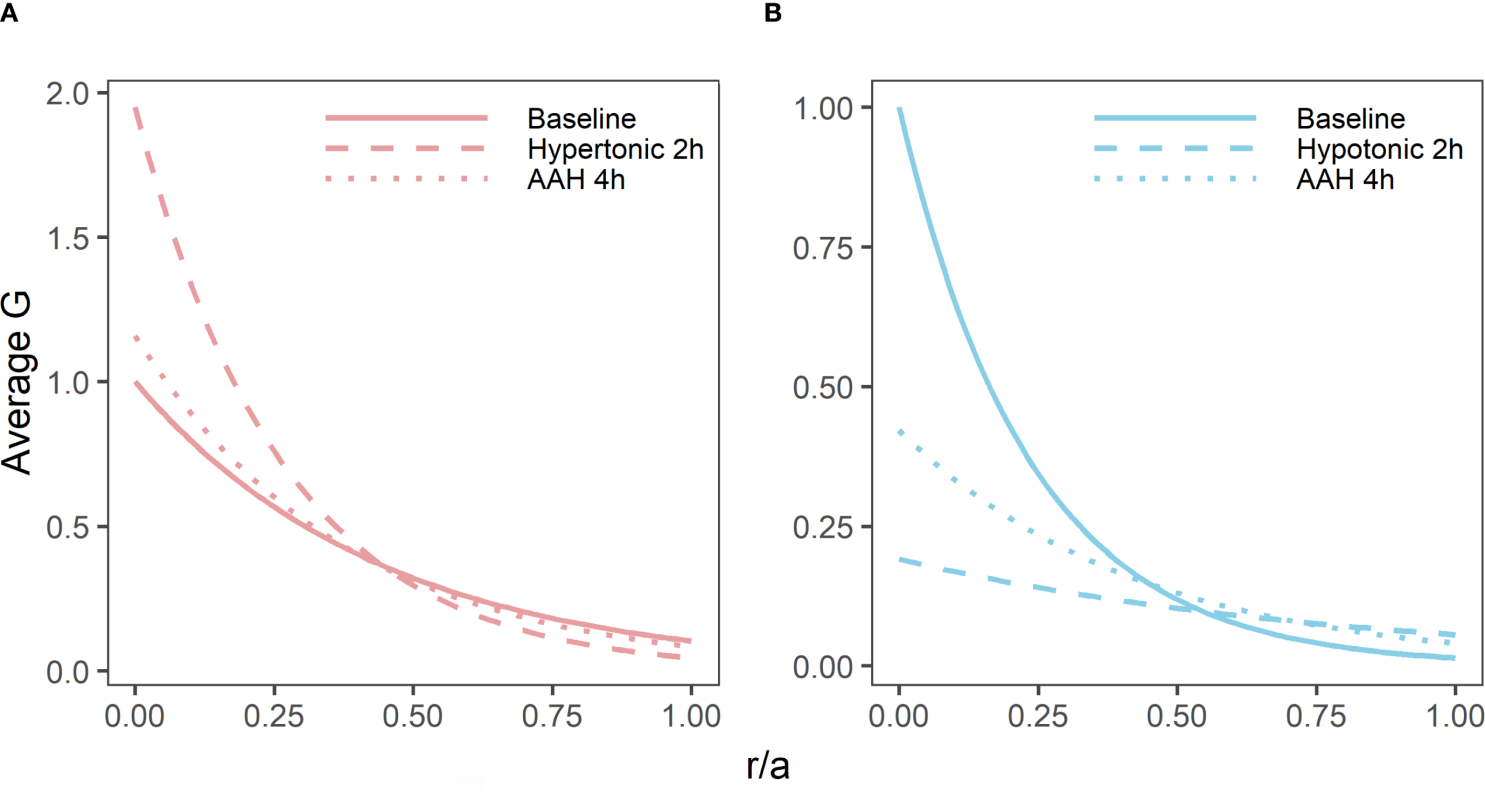
Reversibility of the effects of osmotic challenge on the shear modulus proline in the bovine lens. Normalised average shear modulus (G) profiles of bovine lenses incubated in isotonic AAH for 4 hours (**A, B**, solid lines) is compared to the effects of incubating lenses in either hypertonic (**A**, n = 3) or hypotonic (**B**, n = 3) AAH for two hours (dashed lines) before returning the lenses back to AAH (dotted lines) for a further 2 hours. Note, while the effects of hypertonic challenge on the shear modulus profile is completely reversible, only a partial recovery is observed for hypotonic treated lenses within the 2-hour recovery period tested in these experiments. All profiles are plotted against normalised lens distance (r/a) where 0 is the centre and 1 is the periphery of the lens.

As well as having different effects on lens water content, hypertonic and hypotonic challenge also had opposite effects on the shear modulus profile (**Figure 5**). While incubating lens in AAH for 4 hours did not change the overall shape of the profile (**Figure 5A**), it did cause a slight decrease in overall shear modulus, which was not statistically significant (**Figure 5D**). In contrast, exposure to hypertonic challenge caused a localised increase in the shear modulus in the lens nucleus (**Figure 5B**), which was deemed to be significantly different (**Figure 5E**). In contrast, exposure to hypotonic challenge caused a reversal of the profile (**Figure 5C**), with significant decreases and increases to the shear modulus in the nucleus and cortex, respectively (**Figure 5F**). To determine whether these different effects of osmotic challenge on shear modulus in the different regions was reversible and not due to non-specific structural damage to the lenses, lenses were incubated in either hypertonic or hypotonic AAH for 2 hours and then returned to AAH for a further two hours with shear modulus measurements being collected at the end of each incubation period (**Figure 6**). Lenses exposed to hypertonic challenge exhibited the expected initial increase in shear modulus in the nucleus, which was completely reversed by returning lenses to isotonic AAH (**Figure 6A**). While for lens exposed to hypotonic challenge the observed decrease in shear modulus in the nucleus was only partially ameliorated by the return of the lenses to isotonic AAH (**Figure 6B**).

Taken together these results show that the application of a non-physiologically osmotic challenge, which changes water content in the different lens regions (**Figure 4**), also reversibly alters the shear modulus profile in different regions of the lens (**Figures 5**, **6**). This raises the question as to whether the physiological regulation of lens water content under isotonic conditions via modulation of the lens microcirculation system can also modulate the biomechanical properties of the bovine lens.

### Effect of inhibiting water transport on the shear modulus in different lens regions

**Figure 7:**
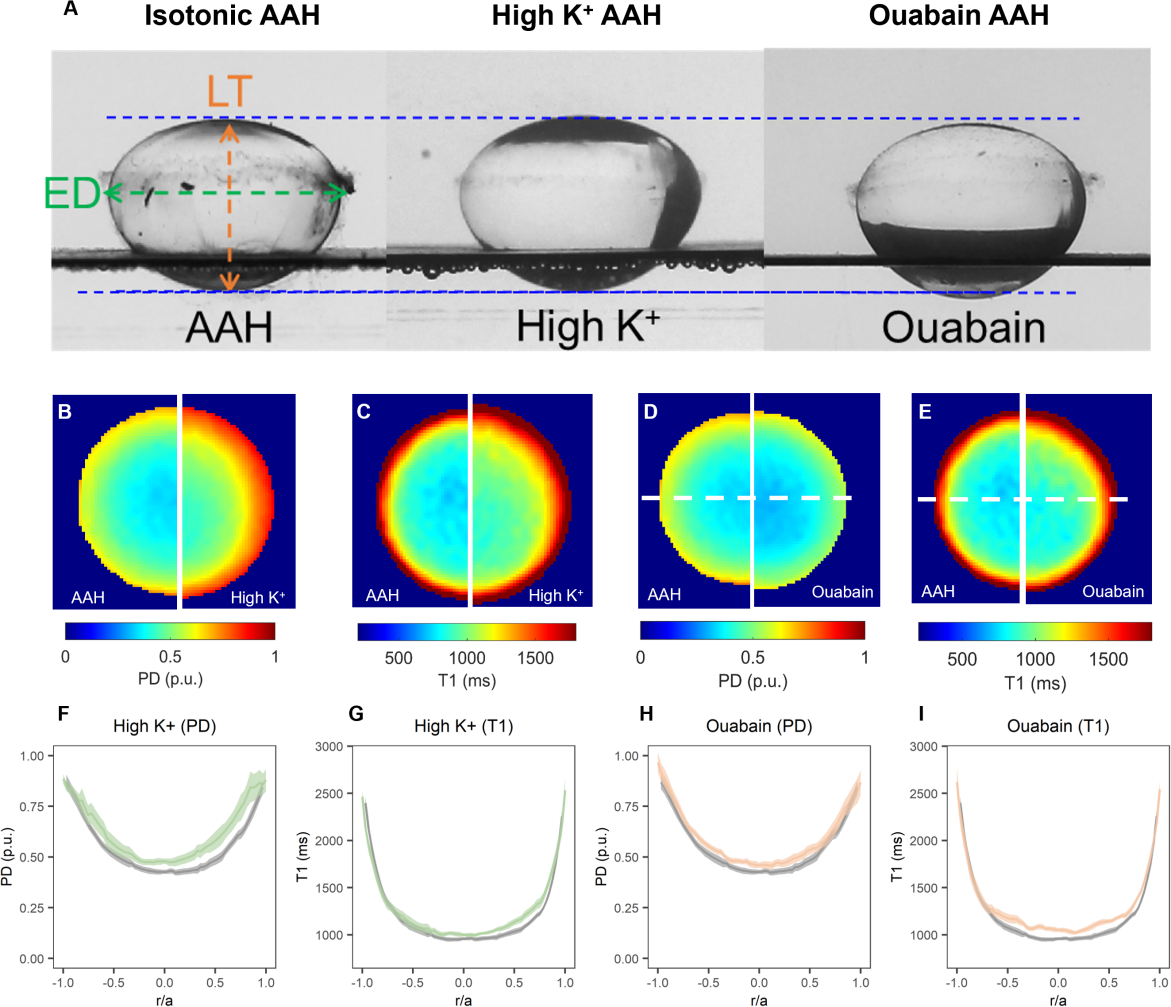
Effect of inhibiting the microcirculation system on total and free water content in organ-cultured bovine lenses. **(A)** Images showing axial views of bovine lenses incubated in isotonic AAH (left), high extracellular K^+^ (middle) and AAH + ouabain (right) showing the changes in lens thickness (LT) and equatorial diameter (ED) induce by the inhibition lens water transport. **(B–E)** Colour maps of total water (PD, **B, D**) and free water (T1, **C, E**) content from representative lenses before organ cultured in AAH (left panels) and after a 4-hour incubation in either high extracellular K^+^ (**B, C**, right panels) or AAH + ouabain (**D, E**, right panels) solutions. **(F–I)** Profiles of taken through the optical axis showing the change total water (PD, **F, H**) and free water (T1, **G, I**) content plotted against normalised lens distance (r/a). Profiles are the average of at least 6 lenses cultured in AAH (grey) followed by a 4-hour incubation in either high extracellular K^+^ (**F, G**, green) or AAH + ouabain (**H, I**, orange) solutions. Error bars indicating the standard deviation.

**Figure 8:**
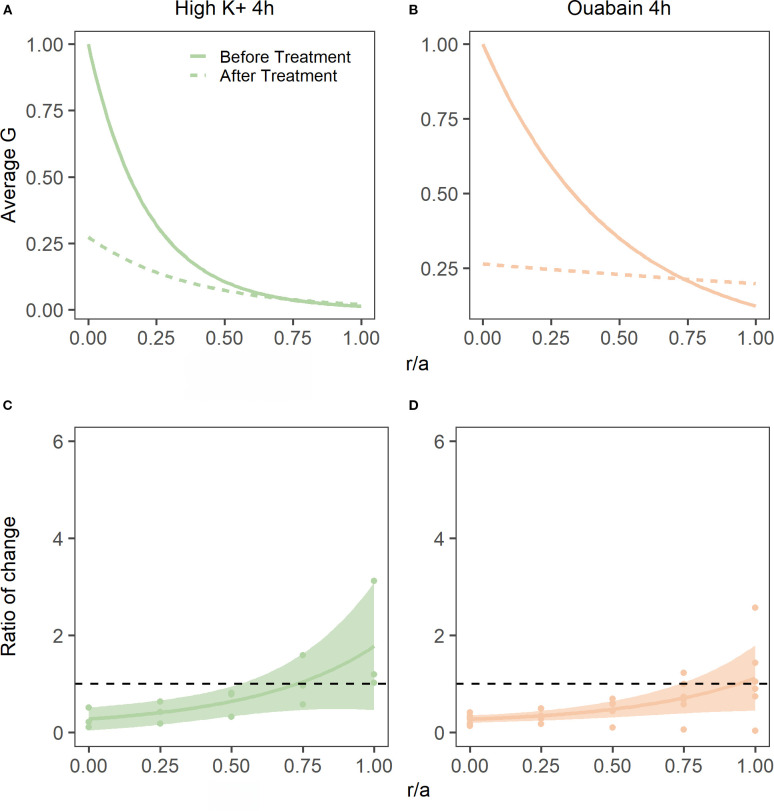
Effect of inhibiting lens water transport on shear modulus profiles in the bovine lens. **(A, B)** Normalised average shear modulus (G) profiles of bovine lenses initially incubated in isotonic AAH (solid lines) and then after (dashed lines) a 4-hour incubation in either in high extracellular K^+^ (**A**, n = 3) or AAH + ouabain hypertonic (**B**, n = 5). **(C, D)** Spatial comparison of regional lens stiffness across the nucleus-to-periphery axis between baseline and post-incubation conditions for lenses incubated in high extracellular K^+^ AAH **(C)**, or ouabain + AAH **(D)**. The dotted line represents no change between conditions, while the shaded regions represent 95% confidence intervals, and the dots are the biological replicates. In all plots profiles are plotted against normalised lens distance (r/a) where 0 is the centre and 1 is the periphery of the lens.

To address this question, we incubated bovine lenses in either high extracellular K^+^ to depolarise the lens potential, or AAH supplemented with 1 mM ouabain to block the Na^+^K^+^-ATPase, two protocols known to inhibit the circulating ionic and water fluxes (12, 33) that generate the lens microcirculation system. Incubation of lenses in either High K^+^ or ouabain both had no effect on lens transparency (20), but did cause changes to overall lens shape (**Figure 7A**, **Table 3**) and lens water content (**Figure 7B**). Interesting, the change in lens water content was most apparent in the lens nucleus (**Figure 7C**), which was consistent with previous MRI studies that also showed that this increase in water content in the nucleus altered the refractive power of the bovine lens (20). This inhibition of the microcirculation by either incubating lenses in High K^+^ (**Figure 8A**) or ouabain (**Figure 8B**) for 4 hours, both significantly reduced (**Figures 8C, D**) the shear modulus in the nucleus of the lens but had minimal effects on the shear modulus in the outer cortex of the lens. Taken together, our results show that increasing the water content of lens nucleus either by swelling the lens by exposure to a hypotonic solution or by reducing the removal of water from the nucleus by inhibiting the microcirculation can both specifically reduce the shear modulus in the lens nucleus.

## Discussion

Based on *in vivo* MRI experiments performed on human lens (9), we have advanced a working hypothesis that changes to both the dynamic and steady state regulation of lens water content maybe an underlying cause of an increase in nuclear stiffness observed in the human lens as it ages and becomes presbyopic. In this study we have taken the first step towards testing this hypothesis by establishing the relationship between lens water content and lens stiffness. To achieve this, we optimised our MRI protocols developed to study water content in the human lens *in vivo* (10, 34), to measure changes in water content in multiple organ cultured bovine lenses exposed to a variety of perturbations designed to alter lens water content. In parallel to these measurements, we adapted a spin test protocol developed by others (21–24), to measure how the stiffness profiles of non-decapsulated bovine lenses are affected by changes to their water content. These proof of principle experiments performed on bovine lenses showed that altering lens water content, via either osmotic challenge (**Figure 4**) or inhibiting lens water transport (**Figures 7**), could indeed alter lens stiffness particularly in the central regions of the bovine lens (**Figures 5**, **8**). We will first discuss the technical limitations of our experimental approach before addressing the biological relevance of our findings in the non-accommodating bovine lens to the human lens.

Multiple approaches have been used to measure biomechanical properties of the lens but many of these were either invasive, in that they utilised lens sections (7, 8) or decapsulated lenses (21–24), and hence compromised the physiological regulation of water transport that we are trying to correlate to lens stiffness. Alternative approaches that preserve the physiological integrity of the lens, such as the application of compressive loads or actuator squeezing on intact lenses (35, 36) have enabled axial and radial strains to be measured but unfortunately do not provide any information on differences in stiffness between the different regions of the lens which exhibit differences in local water content. Regional measurements of lens stiffness can, however, be obtained by spinning lenses to deform the lens and then using a biomechanical modelling approach to quantify the deformation to extract the shear modulus profile across the whole lens (21, 22, 24).

The initial implementations of the spin test were first applied to de-capsulated lenses (21, 22, 24), since it was correctly reasoned that the decapsulation of the capsule would eliminate or substantially reduce systematic errors caused by the constraining effect of the lens capsule (22). Although decapsulation of the lens offers a substantial advantage in terms of solid mechanics modelling, since only the fiber cell mass instead of the combined biomechanics of the fiber cells and the capsule needs to be modelled, the removal of the capsule compromises the biological integrity of the tissue. Hence, in this study we have adopted the spin test as our approach to measure regional differences in lens stiffness, but have applied it to lenses with intact capsules to preserve their physiological integrity so that we can experimentally manipulate their water content. To facilitate this, we have implemented a post-imaging analysis framework (**Figure 1**) that accurately extracts the more restrained change in lens geometry induced by spinning non-decapsulated bovine lenses (**Figure 3**) and used a commercially available modelling platform to estimate regional differences in shear modulus (**Figure 2**).

While our approach allows the physiological integrity of the lens to be maintained, a drawback of modelling the capsule and fiber cell mass as a single entity is an inability to directly compare our results with the values obtained for lens stiffness in other studies in which the lens capsule was removed. Hence, it is not surprising that the values measured for shear modulus in non-decapsulated lenses (**Figure 3**) were some 2–3 times higher than values reported for decapsulated lenses (30). Despite these differences in the absolute magnitude of lens stiffness, the shear modulus profile was essentially similar between intact and de-capsulated lenses bovine lenses, with the nucleus of the bovine lens being consistently stiffer than the outer cortex (30). Moreover, the goal of this study was not to determine the absolute values for lens stiffness. Rather it was to determine whether experimental perturbations designed to alter lens water could induce changes to the shear modulus profile across the lens. Therefore, we were more interested in the relative change in the normalised shear modulus profile induced by a specific perturbation to lenses that retained their capsule and hence could be subjected to organ culture under different experimental conditions.

To determine whether changes in water content can affect lens stiffness we exposed lenses to osmotic challenge and used MRI and the spin test to map the induced changes in regional water content (**Figure 4**) and how those changes affect the shear modulus profile across these different lens regions (**Figure 5**). While organ culturing the bovine lens in isotonic AAH for four hours did not significantly change water content (data not shown) or the shear modulus profile (**Figures 5A, D**), incubating lenses in either hypertonic or hypotonic AAH to shrink or swell the lens, respectively, had significantly different effects on both water content and the shear modulus profile. Hypertonic exposure for 4 hours caused a decrease in the free water content (**Figure 4G**), and a reversible increase in shear modulus (**Figures 5B**, **6A**) which was most pronounced in the central regions of the lens. In contrast, the increase in lens water content seen upon exposure to hypotonic challenge (**Figure 4**) caused a reversal of the shear modulus profile with the nucleus being stiffer nucleus than the outer cortex (**Figure 5C**), a change which was partially reversed by returning the lenses to isotonic AAH (**Figure 6B**). A similar softening of the nucleus (**Figure 8**) was also observed under isotonic conditions by incubating lenses in either high extracellular K^+^ or ouabain (**Figure 7**) to inhibit the ability microcirculation system that actively removes water from central region of the lens. Taken together these experiments show that the stiffness profile of the bovine lens can be manipulated by altering the water content of the lens.

It is interesting to speculate on the relevance of our experimental findings in on the relationship between lens water content and stiffness obtained in bovine lenses to our understanding of the age-related changes in stiffness and water content observed in the human lens. In the human lens the shear modulus profile has been shown to change as a function of age, with the central regions of young human lenses that can accommodate being less stiff than the more peripheral outer cortex (7, 8, 37). However, with advancing age the shear modulus of the nucleus and cortex both increase, but the shear modulus of the nucleus increases more rapidly than the cortex, with the consequence being that from about the age of 45 years onwards the nucleus is stiffer than the cortex (37). Thus, the shear modulus profile of the bovine lens is more reminiscent of an older presbyopic human lens that has lost its ability to accommodate. Our observations that modulation of the water content (**Figures 5C**, **8**) can alter the stiffness profile of the bovine lens opens the possibility that pharmacological modulation of the microcirculation system could be used as a mechanism to regulate lens stiffness in the presbyopic human lens and restore its ability to accommodate.

In support of this contention, we have shown that in young adults, the free water distribution in the anterior lens increases with accommodation, while in presbyopic subjects it decreases when they attempt to accommodate (9). While more recently we have shown that the application of pilocarpine, a reagent that has been promoted to improve near vision by inducing the pinhole effect, also caused free water to become more smoothly distributed across the anterior region of the presbyopic human lens (9). So, while these *in vivo* human experiments show that changes in the distribution of water content are involved in driving the changes to the internal lens composition that contribute to the increase in lens power associated with accommodation (9), they do not directly show that these changes alter the stiffness of the lens nucleus. This caveat is critically important since we have also shown an age-dependent increase in free water occurs in older and stiffer human lenses (10). Hence this previous observation appears to be at odds with our current finding that increases to lens water content induced by experimental perturbations act to reduce the stiffness of the nucleus of the “presbyopic” bovine lens. Since in the human lens it was the free water content and not the total water content that changed with age, it suggests that it is the binding of water to the proteins in the lens nucleus that may in fact be changing and causing the observed age-dependent stiffening of the human lens. Regardless of the molecular mechanism our experiments on bovine and human lenses both highlight a role for water content in setting the stiffness of the nucleus of the lens.

In summary, our proof of principle experiments on bovine lenses have shown that the stiffness of the lens nucleus can be altered by modulation of lens water transport. While it is not yet clear what the underlying mechanism responsible for this change in lens stiffness is, our results suggest that modulation of lens water transport in the human lens may represent a potential strategy to discover novel therapies to restore accommodative capacity in the presbyopes.

## Data Availability

The raw data supporting the conclusions of this article will be made available by the authors, without undue reservation.
